# First in‐vivo magic angle directional imaging using dedicated low‐field MRI

**DOI:** 10.1002/mrm.30332

**Published:** 2024-10-20

**Authors:** Mihailo Ristic, Karyn E. Chappell, Harry Lanz, John McGinley, Chinmay Gupte, Dimitris Amiras

**Affiliations:** ^1^ Department of Mechanical Engineering, Faculty of Engineering Imperial College London London UK; ^2^ Department of Surgery and Cancer Faculty of Medicine, Imperial College London London UK; ^3^ Department of Radiology Imperial College NHS Healthcare Trust London UK

**Keywords:** collagen, magic angle effect, musculoskeletal imaging

## Abstract

**Purpose:**

To report the first in‐vivo results from exploiting the magic angle effect, using a dedicated low‐field MRI scanner that can be rotated about two axes. The magic angle directional imaging (MADI) method is used to depict collagen microstructures with 3D collagen tractography of knee ligaments and the meniscus.

**Methods:**

A novel low‐field MRI system was developed, based on a transverse field open magnet, where the magnet can be rotated about two orthogonal axes. Sets of volume scans at various orientations were obtained in healthy volunteers. The experiments focused on the anterior cruciate ligament (ACL) and the meniscus of the knee. The images were co‐registered, anatomical regions of interest (ROIs) were selected and the collagen fiber orientations in each voxel were estimated from the observed image intensity variations. The 3D collagen tractography was superimposed on conventional volume images.

**Results:**

The MADI method was successfully employed for the first time producing in‐vivo results comparable to those previously reported for excised animal specimens using conventional MRI. Tractography plots were generated for the ACL and the menisci. These results are consistent with the known microstructure of collagen fibers in these tissues.

**Conclusion:**

Images obtained using low‐field MRI with 1 mm^3^ resolution were of sufficient quality for the MADI method, which was shown to produce high quality in‐vivo information of collagen microstructures. This was achieved using a cost effective and sustainable low‐field magnet making the technique potentially accessible and scalable, potentially changing the way we image injuries or disease in joints.

## 1 INTRODUCTION

MRI is commonly undertaken as an imaging modality for diagnosing conditions in joints, owing to its excellent soft tissue contrast and lack of exposure to ionizing radiation. However, conventional MRI struggles with the imaging of ligaments, tendons, menisci, and articular cartilage, which largely consist of extracellular structures of highly organized collagen fibers. As much as 89% of the water present in these tissues is bound to collagen,^1^ making the unaveraged dipolar interactions of proton nuclear spins to be the dominant signal decay mechanism. This results in very short $T_{2}$ and $T_{2}^{*}$ relaxation times, low signal returns, and inherently low contrast.^2^, ^3^, ^4^ Diagnostic imaging of these structures is usually based on a negative contrast with adjacent t, variable amounts of mostly extracellular water in the form of local edema, hemorrhage, or joint fluid. However, reliance on the signal from these fluids to depict damage to the actual collagen structures leads to severe limits in the diagnostic accuracy.^5^ Conditions such as partial ligament tears and chronic injuries remain difficult to diagnose accurately and the accepted diagnostic gold standard is arthroscopy or open surgery, though these methods can be associated with complications and they are generally undertaken only as part of treatment.^5^, ^6^, ^7^

In addition, these tissues exhibit strong field‐related anisotropy associated with the magic angle (MA) effect,^2^, ^3^, ^4^, ^8^, ^9^, ^10^, ^11^, ^12^, ^13^, ^14^ whereby the amount of dipolar coupling is significantly reduced if the collagen fibers are oriented to the main field $B_{0}$ at an angle $\theta \approx 55^{{}^{\circ}}$. Under these conditions, $T_{2}$ may be significantly extended and result in a significant, and often unexpected, increase in the observed signal. From the clinical perspective MA has been viewed as a source of artifacts, potentially leading to erroneous diagnosis of injury or disease. Healthy ligaments and tendons are expected to return low signal (dark), and any brighter regions may be caused by tissue damage or potentially by the MA effect. Therefore, efforts are usually made to minimize the MA effect^15^ by extending the echo time $T_{E}$. The MA effect continues to be a subject of interesting new research related to its impact on MR of tissues such as articular cartilage,^16^, ^17^, ^18^ while improved theoretical models for anisotropic $T_{2}$ relaxation mechanisms are also being sought.^19^, ^20^

Our work set out to exploit the field related anisotropies, and specifically the MA effect, as a potential source of valuable new information about the collagen tissue microstructure. By obtaining images at different $B_{0}$ orientations to the body, we can analyze the recorded intensity variation in tissues exhibiting the MA effect. The recorded intensities can be correlated with theoretical relationships to deduce the dominant collagen fiber orientation in each voxel, ultimately leading to 3D tractography data that can be further visualized and analyzed to assess for function.

Changing the $B_{0}$ orientation relative to the patient is largely impossible in conventional closed bore scanners, and hardly any easier in conventional open scanners. For this reason, we set out to develop a dedicated low‐field MRI system based on a novel transverse field magnet concept,^21^, ^22^ together with the required imaging and computational methods. We have focused on extremity imaging, with the knee as the main initial focus because it represents one of the most challenging and potentially rewarding applications of this method. Imaging other joints such as ankle, elbow and wrist can also be performed. We have previously reported the design of the new magnet system,^21^ and we have also demonstrated the technique of MA directional imaging (MADI)^23^ which was applied to excised animal samples, which were imaged using a conventional high‐field MRI system.

In this paper, we report the first three in‐vivo human imaging results obtained using this dedicated low‐field MRI system with 3D collagen tractography and other plots to display the collagen microstructure. We consider this prototype low‐field MRI to provide an optimal way of performing the MADI method, and we aim to demonstrate its practical viability.

For completeness, the next sections present the MRI system and the implemented software methods, which includes certain improvements not reported previously, before proceeding to present the details of this particular study.

## 2 METHODS

### 2.1 MA MRI scanner

**FIGURE 1:**
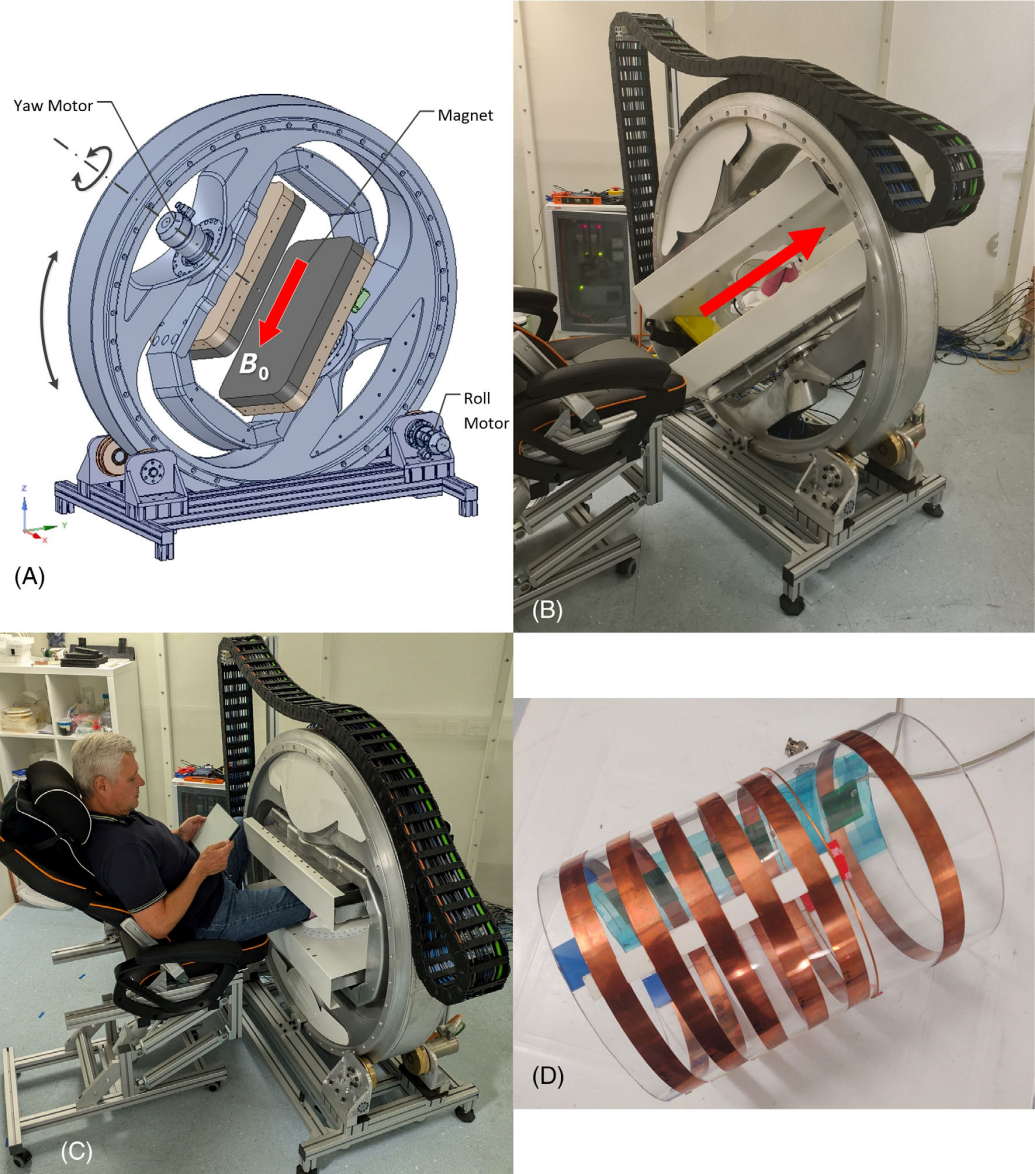
MA MRI, dedicated to extremity imaging, employing low‐field, transverse field open magnet and rotation about two orthogonal axes (*roll* and *yaw*). (A) CAD model showing the magnet arrangement and 2 degrees‐of‐freedom actuation. (B) Magnet in an arbitrary position with $B_{0}$ direction indicated. (C) Magnet and volunteer during scanning. (D) Construction of the solenoid receiver consisting of five inductively coupled resonator loops. The resonator is tuned to 6.4 MHz as the frequency of the first resonant mode, and it is inductively coupled to the preamplifier and detuning circuitry.

The MA MRI system is based on a unique new transverse‐field open 0.15T magnet^21^ shown in Figure 1, consisting of two rectangular 650 × 350 mm pole pieces, with a 15 cm diameter imaging volume, and 23 cm pole gap. Each pole piece comprises an array of permanent magnets representing a North and a South pole, with the return flux provided by the backing plate. The symmetrical arrangement of the magnet results in the field $B_{0}$ being parallel to the pole pieces, while the magnet arrays and a passive shim set have been optimized to provide the required high field uniformity in the central imaging volume.

The pole pieces are carried by a motorized aluminum structure which can rotate the magnet about two orthogonal axes (“*roll*”and “*yaw*”) so the field $B_{0}$ can take any desired orientation relative to a stationary subject. The *roll* degree of freedom has a full 360° range of rotation. The *yaw* degree of freedom is constrained only by the internal hard and soft limits of the positioning system, and it has been set to an allowed a range of ±60° in consideration of system ergonomics during limb imaging. The rotations are powered by an actuation system employing DC servomotors and drives (Harmonic Drive, Limburg, Germany) of the type commonly used in industrial robots. Position feedback is provided by integrated high‐resolution absolute encoders. The magnet was installed in a purpose‐built RF shield within the laboratory. The positioning of the magnet is controlled by a supervisory computer communicating with the intelligent drives inside the shielded room via an optical digital interface. The control system includes various safety interlocks, position, speed and torque limits, and emergency shutdown mechanisms to provide the highest level of safety during operation.

The pole‐mounted gradient set provides 20 mT/m, powered by standard gradient amplifiers (Performance Controls Inc., Montgomeryville, Pennsylvania, USA). Imaging is performed using commercial MRI spectrometer (EVO, MR Solutions, Guildford, UK) providing a wide range of imaging sequences and other usual functionality through a conventional user interface.

The RF system involves separate transmit (Tx) and receive (Rx) coils. Two alternative Tx coils are provided: a pole mounted Helmholtz pair and a saddle coil set integral with a specially designed insert within the magnet. In the latter case, the insert carrying the transmit coil also serves as a guide and an aid for centering the extremity in the magnet, being mounted on the pole covers and free to rotate about the *yaw* axis.

In terms of the RF receiver, the results in this work were obtained using a solenoid‐type coil consisting of a set of five inductively coupled resonant loops (Figure 1D) which were tuned such that the first resonant mode matches the operating frequency (6.4 MHz) of the scanner. A further inductively coupled loop^24^ links the MR signal to the preamplifier and spectrometer through matching circuitry. During the transmit pulse, the receive coil is detuned using a single PIN diode, integrated with the tuning circuit, under control from the spectrometer.

The use of the solenoid receiver does raise questions about the signal strength at various magnet positions. Since the receive coil is stationary together with the subject, while the magnet may be rotated by arbitrary angles about the two axes, the optimal signal strength is achieved when the axis of the solenoid is perpendicular to $B_{0}$. With the present arrangement, the solenoid is clearly not affected by the “roll” angle of the magnet, while the signal will drop with the cosine of the “yaw” angle. This cosine dependence was confirmed in separate studies involving a standard ACR phantom, but it was not found to be a serious limitation in many situations. *Yaw* angles as much as ±30° were found to produce a sufficient signal (intensity degradation of up to 14%), which can be readily compensated by scaling image levels in a pre‐processing stage. In the present study, we limited the yaw angles to ±20°.

### 2.2 Magic Angle Directional Imaging (MADI)

The MA effect was first observed by Berendsen^2^ and later explained by Fullerton.^1^, ^8^ At physiological hydration levels 89% of water in collagen rich tissues may be closely bound to collagen molecules, causing the water molecules to be oriented transversely to the collagen fiber direction. Therefore, a regular structure of collagen results in a regular structure of water molecules, which become “orientationally restricted” and the dipolar interactions become the dominant signal decay mechanism, generally resulting in a very short $T_{2}$. The strength of the residual dipolar interactions depends on the angle θ between the proton–proton direction and the direction of the magnetic field, and it is governed by the term $\left(3\cos^{2}\theta -1\right)$, which will vanish for $\theta =54.7^{{}^{\circ}}$, commonly known as the magic angle (MA).

The observed MRI signal intensity in collagen tissues may be considered to involve contributions from two proton species, one corresponding to the collagen bound water molecules with an anisotropic θ‐dependent values of $T_{2}^{*}$, and the other corresponding to unrestricted water molecules in gaps exhibiting isotropic values of $T_{2}^{*}$. Measured $T_{2}$ values for tendons, ligaments, and cartilage^4^, ^25^, ^26^ have been shown to be in the range 1 ms < *T*_2_ < 25 ms. Imaging sequence with TE < 37 ms show a marked intensity variation due to the MA effect.^15^

Szeverenyi and Bydder^27^ modeled the image intensity I resulting from MA as:

(1) $$I=A.\exp\left(-B\;{\left(3\cos^{2}\theta -1\right)}^{2}\right)$$

where *A* and *B* are constants chosen such that I matches the corresponding intensities observed in the images. They further explain how the dominant direction of collagen fibers in a voxel can be estimated by correlation of the observed intensity values with Eq. (1).

In our previous work^23^, ^28^, ^29^ we presented the MADI method based on this approach, where we focused on optimizing the number of required scanning directions, accuracy and robustness in the presence of imaging noise.

**FIGURE 2:**
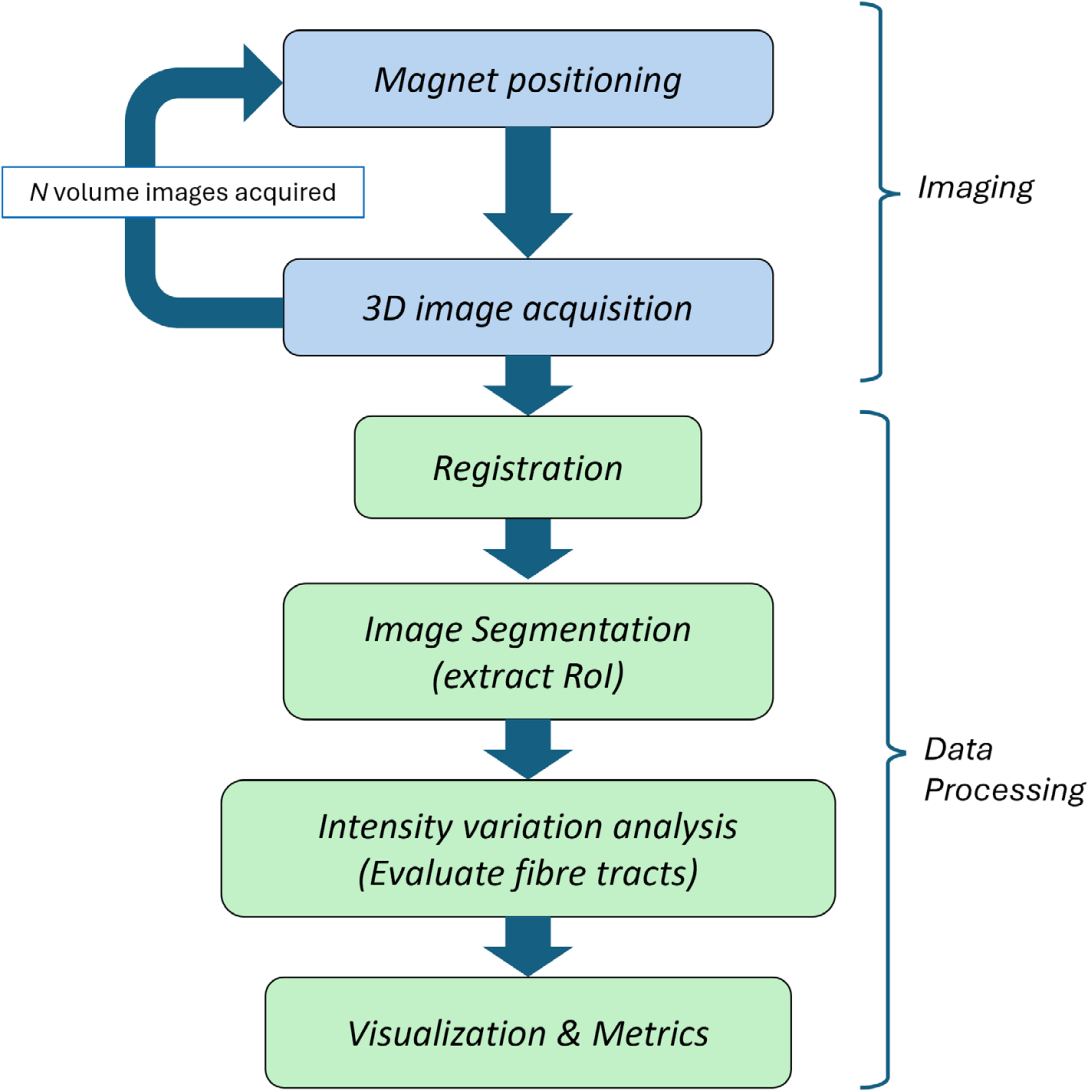
Steps of the MADI process, including MRI imaging and image data processing.

The MADI method is illustrated in Figure 2. It requires obtaining several volume images at various magnet positions, and the overall process may be summarized as follows:

1. *MRI magnet positioning* ‐
  The patient is seated, and the limb being imaged is positioned in the scanner. Before each volume image acquisition, the magnet is first rotated about its *roll* and *yaw* axes to a new desired position, to achieve a desired orientation of the main field $B_{0}$ relative to the anatomy being imaged, while the patient remains stationary in the scanner. Once the magnet positioning is complete, image acquisition can begin.
2. *MRI imaging* ‐
  At each magnet orientation a volume image is acquired. In this work, six magnet orientations were typically employed.
3. *Registration* ‐
  The N acquired volume images are registered to establish voxel correspondences. The final dataset represents a volume image in which each voxel records N acquired intensity values corresponding to N scanning directions.
4. *Segmentation* ‐
  Regions corresponding to tissues that exhibit the MA effect can be identified from the detected changes in image intensity. The ROI corresponding to the anatomy being analyzed may be segmented using interactive tools, and subsequently refined using various metrics, such as SD and range of recorded intensity values for each voxel.
5. *Estimation of collagen fiber direction in each ROI voxel* ‐
  Collagen fiber direction is determined using correlation of the N recorded intensity values with those predicted by Eq. (1), as described below.
6. *Visualization of 3D collagen tractography and quantitative analysis* ‐
  The set of calculated collagen orientations in the set of voxels represents a vector field that can be visualized and analyzed in various ways. It is common to visualize 3D vector field using *glyphs*, or the data may be used to generate tractography plots similar to those used in DTI. Metrics may also be used to quantitatively assess the results, such as using the alignment index^28^ to quantify the level of fiber alignment.

The detailed method presented by Chappell et al.^28^ was modified in this work in several ways, and the details are presented below.

#### 2.2.1 Registration

The registration step was previously performed as a rigid body registration using fiducial markers. This was replaced by soft registration based on image processing methods, consisting of multiple stages. All registration stages use iterative optimization with adaptive stochastic gradient descent, where the metric of Mutual Information (MI) is maximised.^30^, ^31^, ^32^ Using MI helps to align features despite potential intensity differences, rather than simply matching intensities. The MI metric is only evaluated within a sphere of radius 45 mm (using a registration mask) centered within the image volume. Within this spherical region the same information is present across all scans, whereas peripheral regions of some image volumes may omit information due to the edge of the field effects at different scanner orientations.

The iterative registration steps are performed in three stages, as follows:

1. Rigid body transformation
2. Affine transformation
3. B‐spline transformation (soft registration)

The first registration stage starts by using the known *roll* and *yaw* angles of the magnet (telemetry) to bring the two volume images in a sufficiently close alignment in preparation for the subsequent steps. Rigid body registration using MI metric is then performed. The iterations at this stage align the images more precisely than using the *roll* and *yaw* telemetry alone. The second registration stage is an affine transform that uses the rigid registration parameters as the initial condition. This stage allows for some extra flexibility such as shear and scaling of the moving images. Finally, the third registration stage uses a B‐spline transform to correct for any soft body deformations and any geometric distortions due to scanner characteristics. This stage uses the parameters from both previous transformations as its initial condition to compose a single final transform to register each moving image to the fixed image, thus preventing unnecessary blurring from multiple successive registrations. The images obtained experimentally in this work were 150 × 150 × 150 (1 mm voxel size in each dimension). Based on this, the control point spacing was set to 16 voxels (16 mm) in each direction allowing sufficiently fine control over the B‐spline transforms without the need for heavy regularization.

Overall, the rigid and affine stages handle the global aspects of the registration and the B‐spline transform performs final local refinement. To prevent voids being generated during the soft registration step, the transforms are defined from the fixed image to the moving image. A coordinate from the fixed image is transformed into the moving image, the intensity is sampled from the transformed coordinate, and then a resampled image is generated with the sampled intensity rendered at the original coordinate. In this way, voids are avoided while generating a registered image that has structural information from the fixed image, and intensity information from the moving image. The registration steps were implemented using the Elastix toolbox^33^ as the functionality offered was sufficient for our needs.

#### 2.2.2 Estimation of collagen fiber directions

Estimation of fiber direction in a voxel is based on correlation of the measured image intensities with their theoretical variation. As proposed by Szeverenyi,^27^ Eq. (1) can be used to evaluate I using a set of predefined test directions equally distributed over a hemisphere. Each test direction is subjected to the same reorientation of $B_{0}$ relative to the subject in simulation as that during imaging and the corresponding theoretical intensities are computed. The direction that provides the highest correlation (best fit) with the observed values is taken as the estimated fiber direction. This estimate can be further improved by employing it as the initial guess in a subsequent minimization as presented in Chappell et al.^23^ Accuracy and robustness of the method in relation to the number of imaging directions and image SNR were analyzed by Brujic et al.^28^

Focusing now on in vivo imaging using the low‐field scanner, we modified the direction estimation method with a view of further improving robustness in the presence of imaging noise and minimizing the overall scan time. Instead of a set of ˜400 test directions, 50 000 test directions were spread over an entire hemisphere. By reformulating the processing as array operations within MATLAB (The MathWorks Inc., Natick USA), computation time was decreased from ˜1 min to <2 s despite the larger number of test directions. Thus, the optimization step from Chappell et al.^34^ was no longer necessary as the larger number of test directions provided a high‐resolution correlation map to precisely locate the direction with the highest correlation. The most correlated direction was assigned as the estimated fiber direction for each voxel in the ROI. The fiber directions associated with each voxel were then used to perform fiber tractography, similarly to DTI tractography in the brain, so as to model the micro‐structure of the target anatomy.

### 2.3 Imaging studies

The present study involved three healthy volunteers who consented for the study data to be published. Ethics approval for the study was granted by the institution (ICREC ref. 22IC7857).

All images were obtained using 3D FLASH sequence TE = 5.2 ms TR = 13 ms flip angle (FA) = 30° to produce 150×150×150 volume images at 1 mm isotropic resolution with two averages. The scanning time for each volume was about 10 min. TE was chosen to be reasonably short, ensuring that the MA effect is prominent.

As the MADI method involves obtaining several volume images of the anatomy at different magnet orientations, it is desirable to optimize the overall scanning time through a judicial choice of scanning directions. In this, the key factor is that the recorded signal intensity variation adequately correlates with the theoretical relationship of Eq. (1). Our previous work^23^, ^28^ has shown that typically seven or more equispaced scanning directions are needed to detect any arbitrary fiber orientation, while using a priori knowledge of the anatomy^29^ this may be reduced to as few as four in some cases.

In the present work we focused mainly on the knee, primarily the anterior cruciate ligament (ACL) and the meniscus. In view of the constraints imposed by the sensitivity of the solenoid receiver coil in relation to $B_{0}$, we aimed to limit the yaw angle while allowing full range of roll angles of the magnet. The specific choice of scanning directions was guided by this consideration, and by the anatomy obtained from scout scans.

Normally, the first scan is performed with yaw at 0° and roll at either 0° or 90°, and this initial position served as the reference for the sagittal/axial/coronal anatomical planes in relation to the scanner. We typically performed six volume scans in each session, involving roll angles in the range +90° to −60° (where 0° roll corresponds to $B_{0}$ being horizontal) and yaw angles in the range ±20°. As the sensitivity of the solenoid receiver falls off according to the cosine of the yaw angle, the recorded image intensities were normalized accordingly during postprocessing. Prior to each acquisition, the RF center frequency, linear gradient offsets, and RF transmit coil calibration were checked to allow for any drift caused by thermal or magnetic environmental variations and to maintain consistent transmit FA excitation.

The DICOM volume images acquired at different magnet positions were first registered and then manually segmented to extract the ROI using a segmentation mask generated using 3D Slicer Version 5.6.2.^35^ The segmentation was performed only on the fixed image as all other images were aligned to it. The resulting data thus consisted of a set of corresponding voxels within the ROI, each set containing the *N* recorded intensity values at that voxel location. The ROI was further refined by eliminating those voxels showing SD of recorded intensities below a certain threshold, so that the remaining voxels in the ROI are only those considered to exhibit the MA effect and therefore making up collagen fiber structures. Collagen fiber direction vectors were then computed for each voxel. Therefore, the output of the process is a vector field corresponding to the estimated fiber directions in the ROI.

In a basic form, the vector field of computed fiber directions can be displayed using glyphs. For better visual presentation the data can also be used to generate 3D tractography plots. For this, we employed the tractography tools provided by 3D Slicer Diffusion Toolbox. Since these tractography tools were developed to visualize data consisting of tensors (as provided in DTI), whereas our data consist strictly of vectors, the input to the tractography tools was provided by generating synthetic tensors with the principal eigenvector in the computed fiber direction and the other two eigenvectors being assigned arbitrarily small magnitudes.

## 3 RESULTS

**FIGURE 3:**
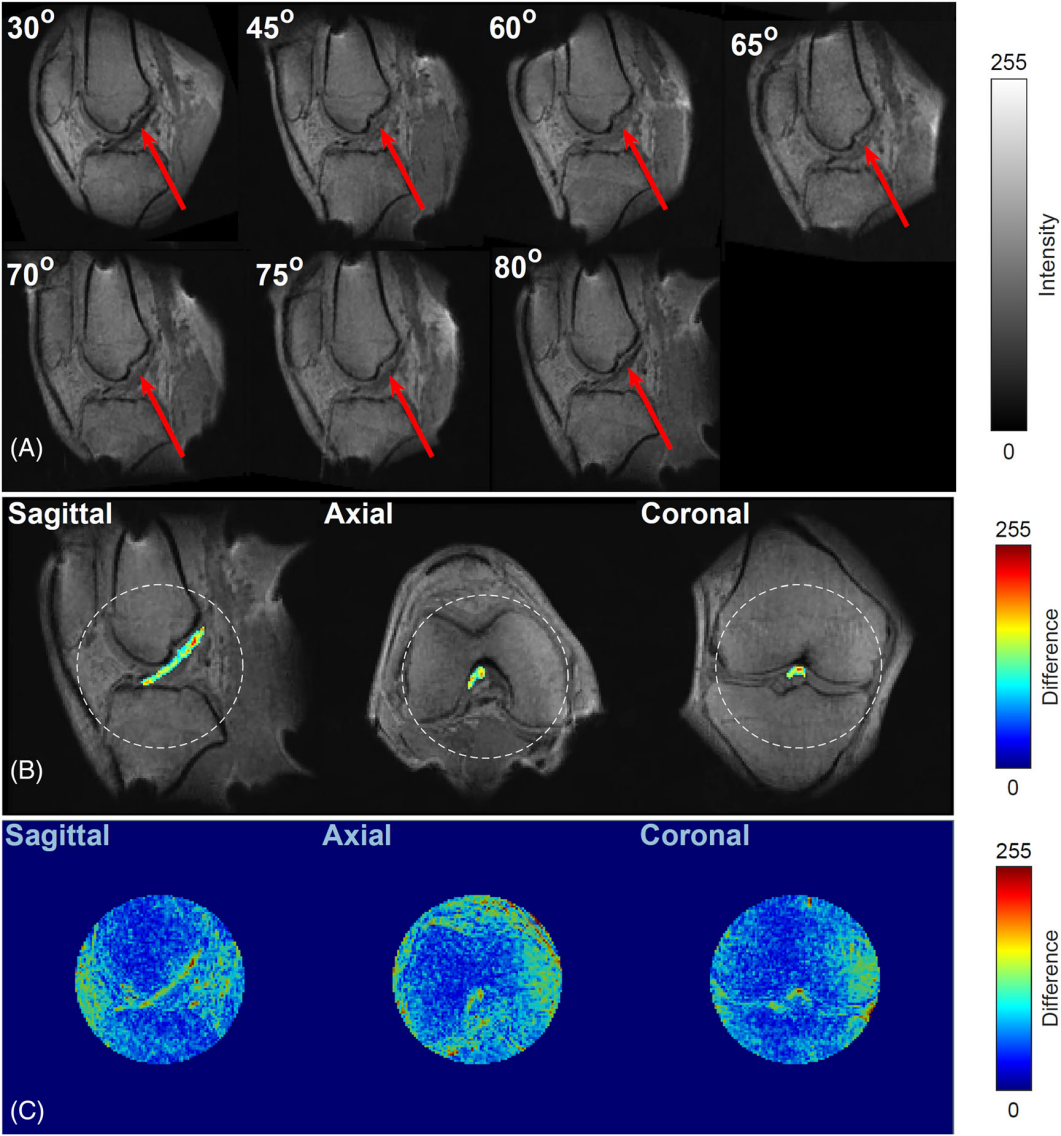
MA effect detected for the ACL using 3D FLASH TR15ms TE4.43 ms FA30°: (A) Images of the ACL (indicated by arrows) at various angles θ relative to the main field *B*_0_. Increased signal is observed near 55°. (B) Subtraction images of a segmented ACL displayed on an arbitrary color scale for easier visualization, superimposed on grayscale images of the knee anatomy. (C) Difference images for the recorded minimum and maximum intensity corresponding to the circles indicated in (B) on an arbitrary color scale for easier visualization.

Figure 3A shows sample 2D sagittal sections, demonstrating the MA effect in the ACL at different orientation of the main field. In each case, the indicated values of angles correspond to the angle θ between the fibers and the main field $B_{0}$ since the collagen fibers in the ACL are predominantly aligned in one direction. The range of observed intensity variation was found to vary for different tissues. These results are made much more apparent in Figure 3B which shows the difference between the maximum and minimum recorded intensity for each voxel in the ROI, superimposed on the conventionally acquired T1w images.

**FIGURE 4:**
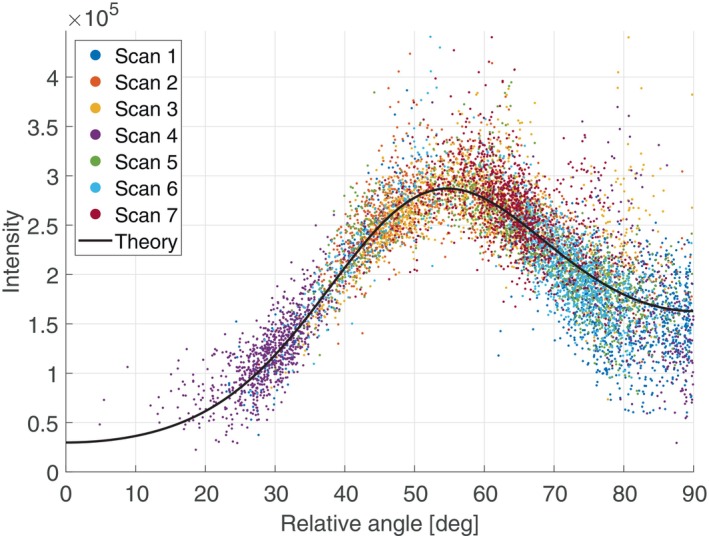
Typical plot of recorded image intensities versus angle θ between $B_{0}$ and the estimated fiber direction for the ROI corresponding to the ACL. Each point corresponds to one voxel and each color corresponds to one magnet orientation relative to the subject.

A typical observed overall intensity variation for ACL is shown in Figure 4. This plot records all intensity values for all voxels in the ROI against the calculated angle θ between the estimated fiber direction and $B_{0}$. The points in the plot are color coded according to their scan orientation. The solid black line shows the theoretical relationship represented by Eq. (1).

**FIGURE 5:**
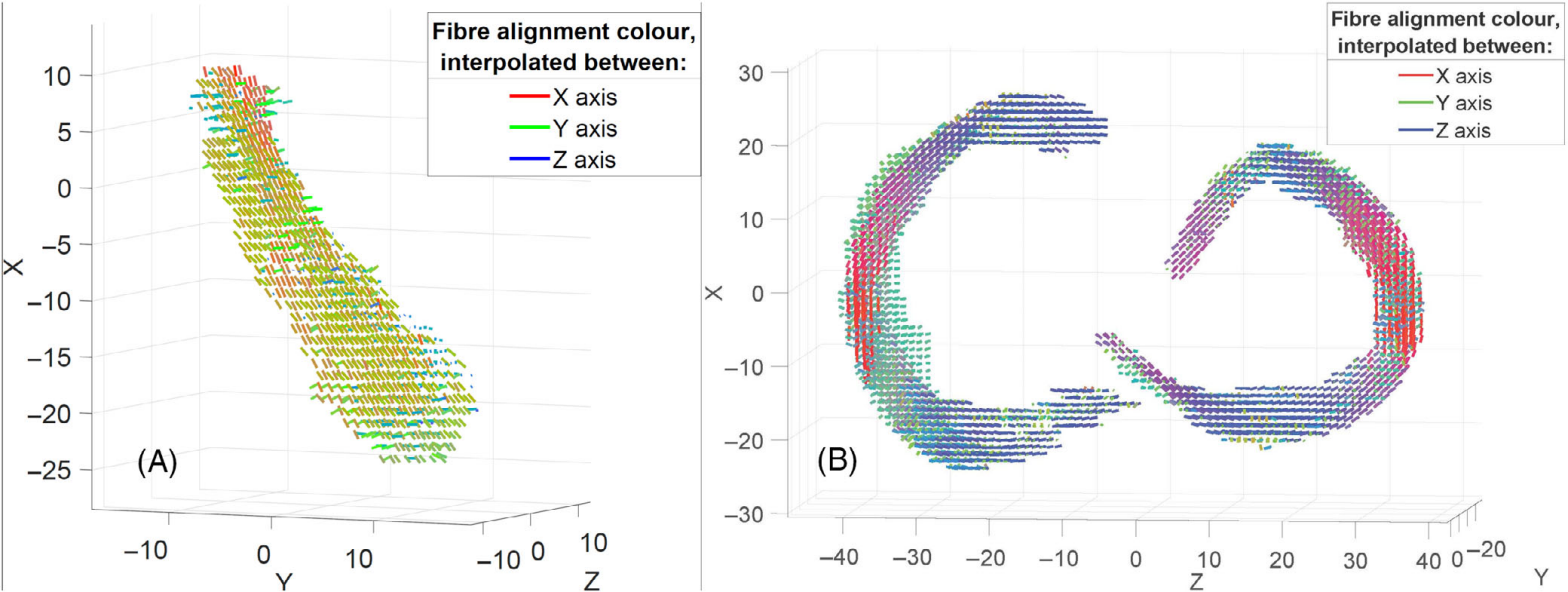
Calculated collagen fiber orientations visualized in a 3D glyph plot and colors corresponding to the orientation relative to the indicated XYZ coordinate frame: (A) ACL and (B) the meniscus.

Figure 5 shows calculated collagen fiber orientations for the ACL (A) and the meniscus (B) displayed as a 3D glyph plot, representing the main result of the MADI process.

**FIGURE 6:**
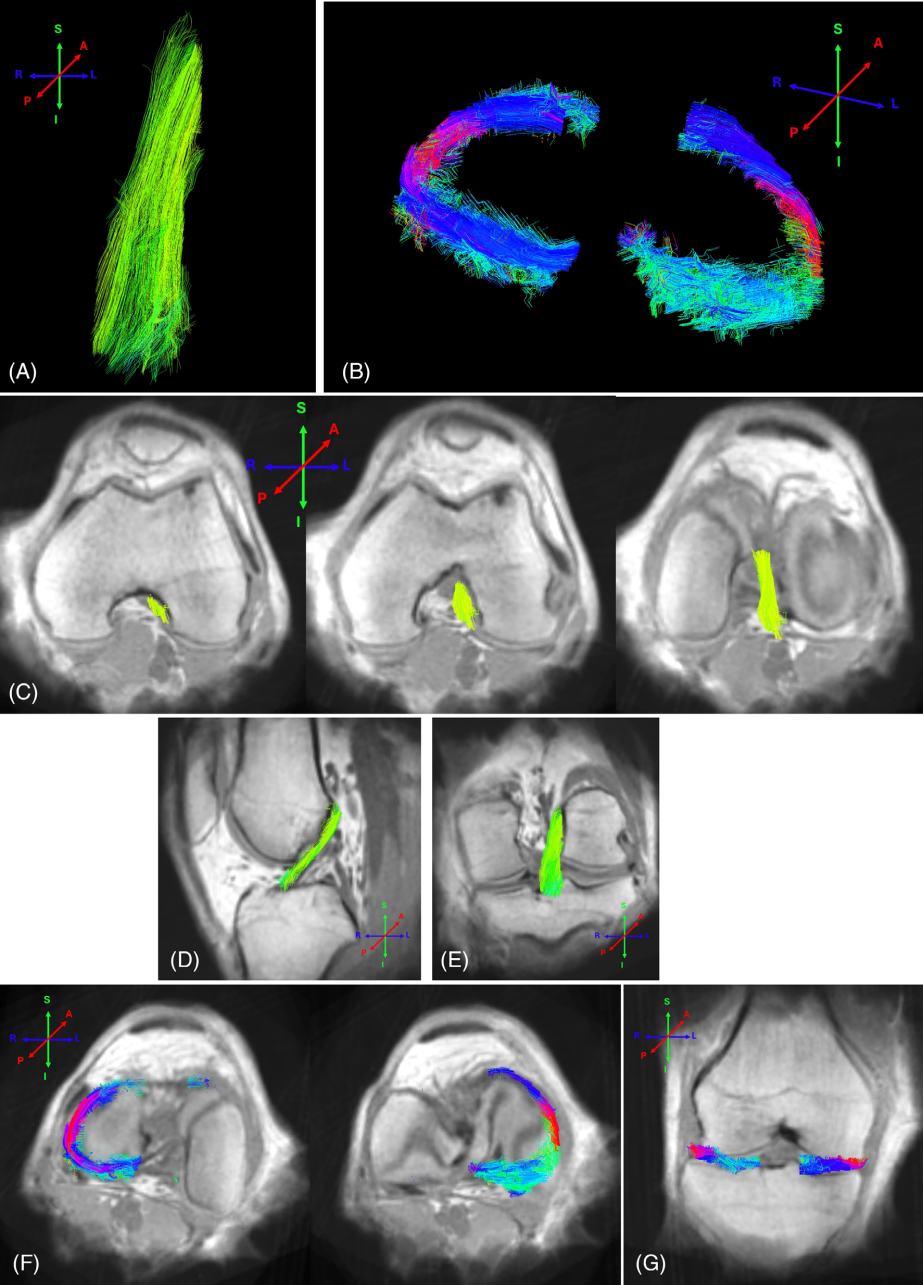
Collagen tractography results with colors indicating orientation relative to the reference axes of the human body (left‐right, anterior‐posterior and superior‐Inferior). The grayscale background images are averages of all volume images acquired at different $B_{0}$ orientations. (A) ACL. (B) Meniscus. (C) Axial view of the 3D tractography of the ACL, visualized in combination with three different axial planes through the knee volume images. (D) ACL sagittal view. (E) ACL coronal view. (F) Axial views of the meniscus with different axial planes through the volume; (G) meniscus coronal view.

Figure 6 shows collagen tractography results for the ACL (A) and the meniscus (B). Figure 6(A–C) show various views of the 3D tractography results in combination with selected planar slices through the overall volume image.

## 4 DISCUSSION

The use of the new transverse‐field open magnet MRI has been found to be both successful and practical, with the volunteer subject sitting comfortably outside the magnet which can be rotated around their limb. Performing MRI scans of the knee in the manner described here is not possible in conventional closed‐bore scanners. Even in the conventional open‐bore scanners this would also be difficult, since the patient may need to be placed in different, and probably uncomfortable, positions, while the changes in orientation of $B_{0}$ would be very hard to control.

The diagnostic value of low field MRI continues to be a subject of some debate, with image SNR and resolution often being the overriding criteria. Despite the considerable success of some commercial systems, the conventional clinical practice typically recommends that extra‐axial musculoskeletal imaging is performed using high field MRI, and 3T systems often seen to be preferable to 1.5T. Indeed, one of the major aspects of our work has been to demonstrate that low‐field MRI (0.15T in this case) can be successfully employed to carry out the MADI method. The experience and results obtained to date are highly encouraging, and in our view demonstrate the practical value of the low‐field system.

Soft registration was found to be a key enabling tool in the MADI method. Low‐field MRI employing permanent magnets and passive shimming is known to produce inferior $B_{0}$ uniformity compared with conventional MRI and therefore more pronounced image distortion. If the body is imaged at different magnet positions, then the geometric distortion of the anatomy in different views will also be different. In our previous work involving imaging of small samples placed at the isocenter of a hospital MRI, it was sufficient to correct the images using the built‐in distortion correction and then to apply rigid‐body registration based on fiducial markers, while in some cases manual fine tuning was also necessary. However, the rigid registration approach was inadequate for human imaging described here. Attempts to achieve sufficiently accurate geometric correction through calibration could not achieve the required accuracies and the procedures were found to be overly complex. The adopted soft registration process was fine tuned to meet the needs of the MADI method, and it was found to provide a fully automatic, flexible, and computationally efficient solution.

Water‐fat in/out of phase issues in the context of low field MRI were considered. In order to produce a pronounced MA effect, the $T_{E}$ value used was short (4.43 ms), while the in/out‐of‐phase times at 0.15T are much longer at ˜22 and ˜44 ms, respectively, so a Dixon technique^36^ could not be applied. Inversion recovery could provide fat suppression, although at this field strength the frequency difference is only 22.4 Hz and the fat/water spectra overlap. Consequently, no fat suppression was used in the results presented here, but this was not an issue in the context of detecting collagen microstructures. Of course, in a clinical setting of MSK examination, fat suppressed images are a required part of the protocol and those could be obtained in addition to 3D FLASH images for the MADI method, using a form of the Dixon technique.

Determining the Ernst angle for low‐field imaging of ligaments posed some challenges, as there is little published data for the relevant $T_{1}$ values. We performed experimental studies to determine the optimal FA, which was estimated to be about 30°, and this was later found to be consistent with the Ernst angle calculated using $T_{1}$ values from Pettersson and Slone.^37^

The recorded intensity values associated with the MA effect depend on the $T_{2}/T_{2}^{*}$ relaxation times, but these were not explicitly considered in the adopted model. The intensity is also influenced by the echo time $T_{E}$ and the FA. The adopted model has the advantage that $T_{1}$ weighted gradient echo images are considerably faster to acquire than quantitative $T_{2}$ mapping, and intensity values are the direct measurements obtained by the scans. Our experience confirmed previous reports^27^, ^28^, ^34^, ^38^ that Eq. (1) is a sufficiently general and robust model, and parameters A and B can be readily tuned to match the recorded data. The correlation used to estimate the fiber orientation was found to be quite robust in relation to the values of A and B, if the selected set of scanning directions adequately captures the function ${\left(3\cos^{2}\theta -1\right)}^{2}$ governed by the MA effect. It should be noted that there has been interesting recent work^19^, ^20^, ^39^ proposing more accurate models for $T_{2}$ relaxation in relation to tissue anisotropy, especially in the context of analyzing collagen structures in the articular cartilage. This could be explored in the future using techniques of quantitative MRI.

It is clearly desirable to optimize the overall scanning time through a judicial choice of scanning directions. Our previous work^28^ has shown that typically seven or more equally spaced scanning directions are needed to determine any arbitrary fiber orientation, but as few as four views may be sufficient if a priori knowledge of the anatomy can be employed.^29^ Nevertheless, in the exploratory work presented here the number and choice of scanning directions were guided mainly by the constraints imposed by the available receiver coil characteristic. Thus, we opted to perform six acquisitions covering a full range of roll positions of the magnet (for which there is no degradation in the receiver signal) and yaw angles within ±20° (signal reduction by up to 6%) with the aim to capture the characteristic dictated by Eq. (1). Although the range of yaw angles was relatively small, this was found to be sufficient to produce robust detection of fiber directions. Also, we conservatively opted for performing acquisitions with two averages, giving around 10 min scanning time for each $150^{3}$ volume acquisition with 1 mm isotropic resolution.

A limitation of the current method is that the solenoid receiver coil design has reduced sensitivity of increasing *yaw* angle, which limits the range of imaging directions that result in adequate signal. Addressing these constraints is expected to bring significant benefits in improving the flexibility and speed of the overall method. The clear challenge here is to simultaneously achieve high sensitivity with a good filling factor (close fit to the body), maintain sensitivity for different magnet orientations, and maintain ergonomic compatibility. Different candidate design involving coil arrays are currently under consideration.

A limitation of this study is that it does not assess the accuracy of the results in relation to an established gold standard. The definitive method of analyzing collagen fiber structure is polarized light microscopy of excised small samples, but this is not compatible with non‐invasive in‐vivo studies and cannot be easily applied to visualize the overall fiber structures of a ligament or the meniscus. Nevertheless, our results were found to be consistent with previous published work. Seidel et al.^38^ analyzed the accuracy of the model of Eq. (1) in comparison with measurements obtained using polarized microscopy. This was further analyzed in our previous work involving Monte Carlo simulations and animal models.^28^, ^34^ The overall observed collagen structures are also consistent with the known anatomy of the ACL^40^ and the meniscus.^41^

The study was also limited to healthy volunteers, and no attempt was made to investigate any pathology.

Our future work will be directed to further improve performance of the MADI method and to provide evidence of its diagnostic accuracy. Further studies are required to validate the improved visualization of collagen fiber orientation in the connective tissue structures of the knee. However, if such validation is achieved, there are several potential clinical applications for such a system:

1. Diagnosis of partial ACL injuries, especially distinguishing between anteromedial and posterolateral bundle tears.
2. Determining more accurately than conventional imaging, the precise pattern of meniscus tears, for example, oblique, horizontal cleavage, or radial tears.
3. Assessing injuries to the chondral cartilage lining the articular surfaces of femur, tibia, and patella of the knee joint.
4. Diagnosing and distinguishing between injuries to the superficial and deep medial collateral ligament.
5. Diagnosing injuries to the anterolateral capsule including the anterolateral ligament and deep portion of the iliotibial band.

With this in mind, plans are under way to conduct clinical studies using this system. Volunteer patients with diagnosed ligament or meniscal injury and scheduled for surgery will be scanned using the system and the findings will be compared to those obtained through both conventional MRI and surgery. A comparison with surgical findings is necessary, since arthroscopy or open surgery are the currently accepted diagnostic gold standard.

In terms of hardware, in the future, we plan to realize improvements in the design of the receiver front end, by investigating coil array designs that are better optimized for this application, to allow practical utilization of a wider range of orientations that the magnet can achieve. In terms of the overall method, we will investigate the use of sparse acquisition and non‐Fourier reconstruction that could lead to significant improvements in scanning time. Combined with the anticipated receiver improvements, we aim to bring the overall required scanning time to about 30 min.

## 5 CONCLUSIONS

We have successfully demonstrated the MA MRI system based on a rotatable low‐field magnet and obtained in vivo volume images of a sufficient quality for their use in the proposed method. We have successfully applied the MADI method to those images to produce highly encouraging collagen tractography results. Prior to this work, the condition of highly aligned collagen structures in MRI were predominately identified by negative contrast using a fluid sensitive sequence or contrast agents such as intra‐articular gadolinium injection to identify defects in known structures such as the ACL. For the first time, we can now image highly aligned collagen structures directly and with further work potentially negating the need for routine fluid weighted sequences for the identification of defects or injuries We believe that application of this method could lead to significant improvement in the diagnostic accuracy for MRI for ligaments, tendons, menisci, and other tissues consisting of collagen structures. Being low‐field, MA‐MRI is inherently low‐cost in comparison with conventional hospital MRI systems, and this could be a significant factor to facilitate its future adoption.
